# Perspectives on human regeneration

**DOI:** 10.1057/s41599-018-0118-4

**Published:** 2018-06-12

**Authors:** James F. Stark

**Affiliations:** 1 University of Leeds, Leeds, UK

## Abstract

Regeneration is a concept that has fascinated humans for centuries. Whether we have been trying to bring things back to life, extract additional resources from the world, or remodel our living spaces—domestic and urban—it is often presented as an unproblematic force for good. But what exactly does it mean to regenerate a body, mind or space? This paper, which introduces a collection of contributions on the theme of human regeneration, explores the limits and possibilities of regeneration as a conceptual tool for understanding the biological realm. What does it mean to be regenerated? How can a scholarly focus on this concept enrich our histories of bodies, ageing, disability and science, technology and medicine? As a secondary goal, I identify two distinct aspects of regeneration—'hard' and 'soft' regeneration—which concern the medical and social elements of regeneration respectively. By recognising that everything from cosmetics and fictions to prosthetics and organs grown *in vitro* display a combination of 'hard' and 'soft' elements, we are better placed to understand that the biological and social must be considered in consort for us to fully appreciate the meanings and practices that underpin multiple forms of human regeneration.

## Epigraph

‘Look there’s a gleam! – Now hope may be fulfilled,
That hundreds of ingredients, mixed, distilled—
And mixing is the secret—give us power
The stuff of human nature to compound
If in a limbeck we now seal it round
And cohobate with final care profound,
The finished work may crown this silent hour.
It works! The substance stirs, is turning clearer!
The truth of my conviction passes nearer
The thing in Nature as high mystery prized,
This has our science probed beyond a doubt
What Nature by slow process organised,
That have we grasped, and crystallised it out.’^1^
— Goethe, Faust, Part II Act 2, ‘Laboratory Scene’

## Introduction

The creation of a homunculus by Faust’s companion, Wagner, constitutes a deep engagement with aspects of scientific practice in the teeth of the industrial revolution. Goethe’s own immersion in a diverse range of subjects, from colour theory and optics to botany and physiology, were a source of inspiration for a laboratory scene, which on an initial reading, appears to resonate with contemporary debates about spontaneous generation and the limits of scientific knowledge and explanatory power (Fink, 1991, pp 24–26, 87–88).^2^ Wagner is able to master Nature in the same way that Robert Koch would do with microorganisms over a century later.

However, we might also explore an alternative interpretation to events in the drama. Instead, let us afford prominence to a rather different scientific theme: not spontaneous *generation*, but that of *regeneration*. As Wagner, watched over by Mephistopheles, rejoices in his success and engages directly with his creation, he reflects on the turgidity of natural biological processes (Nature by slow process organised), the ability of scientific knowledge to first penetrate (we grasped) and then refine (crystallised it out) these mysterious happenings. This is not creation but reorganisation. In many ways these very few lines can be read as a microcosm for the different features of scientific practice identified by John Pickstone in his well-known ‘ways of knowing’ thesis: natural history, analysis, experimentation and technoscience (Pickstone, 2000). However, there are more specific and striking parallels with the diverse field of regeneration research in all its many guises. Wagner was attempting to manipulate life at a fundamental level by treating both body and mind as fundamentally chemical processes, with everything from lifespan (explored, of course, in much more detail elsewhere in *Faust* through his much-studied quest for immortality) to consciousness within the ken of scientific manipulability. These efforts mirror, if not in scale or aim at least in type, modern techniques and tools designed to prolong life and maintain health, particularly in the field of regenerative medicine.

Consideration of regeneration as a specific theme in histories of science has been largely confined to discussion of the work of a small group of leading figures in the biological sciences who were active at the outset of the twentieth century, amongst them such individuals as the American biologist Thomas Hunt Morgan, who himself drew heavily on research carried out over the previous two centuries (Maienschein, 2011). In this paper, and drawing on the contributions across the accompanying article collection, I argue that the broad and far-reaching concept of regeneration underpinned a range of significant and fascinating bio-social innovations, from the development of new cosmetic products and procedures designed to preserve a gendered, racialized and youthful aesthetic to biomedical attempts to reengineer the human. Alongside these, imaginings of regeneration and its counterpoint—degeneration—provided inspiration for works of art and fiction exploring ageing, corporeality and the potential dangers of a mature, successful science of human (and non-human animal) regeneration. Collectively, these drew on the latest scientific research, from hyperbolic claims to grounded realism, as well as long-established utopian and, far more frequently, dystopian perspectives on radically extended lifespans and the ability to fundamentally alter the native human condition.

One of the key conceptual features of 'regeneration' in relation to human beings is its breadth. A helpful framework which we might usefully adopt when approaching the topic is to consider the interaction between professionalised, highly medicalised forms of regenerative practice—what I term 'hard' regeneration—and the everyday, domestic aspect of regeneration, best understood as 'soft' regeneration. When viewed as a spectrum of practice, efforts to regenerate the human body and mind can be located as displaying aspects of both hard and soft practice. For example, softer practices involving cosmetics and skin care, used in the confines of homes and bathrooms, might seem to be detached from 'harder' practices of surgical rejuvenation, yet many advertisements make explicit references to developments in endocrinology or nutrition science in order to persuade consumers of their efficacy. Similarly, the hard regeneration achieved through the use and development of prosthetics and biological scaffolds cannot be properly understood without reference to the 'softer', social aspects of its impact; in this case, the effects of regenerative medicine are ultimately in the everyday lives of patients.

Aspects of both 'hard' and 'soft' regeneration have ancient roots. Core concepts underpinning the modern science of regeneration can be traced to Aristotelean ideas posited in *On Generation and Corruption* (de Haas and Mansfeld, 2004). However, the brand of regeneration research which might be classified as in the same tradition of contemporary regenerative medicine stemmed from ideas articulated at the very outset of the twentieth century. In January 1900 Thomas Hunt Morgan delivered a series of five lectures at Columbia University. In these synoptic expositions of biological science his theme was ‘Regeneration and Experimental Embryology’, a topic that Morgan had been engaged in researching at his home institution of Bryn Mawr College for around 10 years (Morgan, 1901, p vii). These lectures would be published the following year as a book entitled simply *Regeneration*, and Morgan drew on examples of the phenomenon of biological regeneration of cells, tissues and structures from across the natural world, beginning with a historical account of investigations into regeneration and ending with a rebuttal of the atomistic conception of life which stemmed, Morgan argued from ‘[t]he gemmules of Darwin … the intracellular pangens of De Vries, the plasomes of Wiesner, the biophors of Weismann, the idiosomes of Hertwig, and the micellae of Nägeli’ (Morgan, 1901, p 278). Rather than regarding the organism as a series of connected yet autonomous sub-structures, Morgan noted that ‘the most fundamental characteristics of the organism, those that concern growth, development, regeneration, etc., seem to involve in many cases the organism as a whole.’ (Morgan, 1901, p 278).

Much has been made of Morgan’s work, yet he was just one of a significant group of prominent scientists who in the early twentieth century became fascinated by the mechanisms underlying physiological regeneration (Allen, 1978; Kohler, 1994). As well as Morgan, his close friend and associate E. B. Wilson and the Russian zoologist Élie Metchnikoff were just two of a high-profile cluster of researchers who attempted to shed light on the process of biological development by examining parallels with regeneration in nature. Collectively they manipulated organisms and proposed modes of action which had far-reaching scientific and philosophical implications for our understanding of human bodies and their capabilities. The emergence of a form of holism underscored by developmental biology was just one feature of this.

Metchnikoff in particular became fascinated with lifespan in humans and animals. Like many of his contemporaries who found notoriety in this field, he was a credible scientist who achieved widespread recognition in the field of immunology. He was strongly committed not just to understanding the underlying mechanisms and patterns of ageing, but also to exploring how human life might be extended and health maximised (Tauber and Chernyak, 1991). Metchnikoff’s optimism in this regard was largely based on his experience of working with animal models and extrapolating to the human, yet others active in the early decades of the twentieth century were interested not just in imagining the implications of a fully-mature public health but of bringing about rejuvenation—the restoration of youth—by a variety of means. It is the connections between the quest to explain mechanisms of regeneration in nature and the search for viable methods of achieving rejuvenation in human beings which this article brings into focus for the first time.

On the face of it the physiological processes of hydra, sea urchins, and other 'lower' animals that were the main focus of much of Morgan’s work had little in common with the very public debates about extending human longevity which were so characteristic of the interwar period. However, closer examination reveals that the fundamental cellular processes that dominated the science of regeneration in the early twentieth century were also critical in enabling would-be rejuvenators—for the most part fringe figures in medical science, and certainly marginal ones in physiological research—to create plausible narratives for their claims.

For example, in his work *The Conquest of Life* which first appeared in English in 1928, Serge Voronoff, the Parisian surgeon who achieved public notoriety through his rejuvenation experiments, lectures and procedures, argued that the root of the ageing process lay in ‘the predominance of conjunctive cells replacing highly differentiated cells—a veritable triumph of anarchy, an ephemeral reign of inferior elements—from which results the disorganisation of all functions and the final death of the organism’ (Voronoff, 1928, p 27). Voronoff was committed to an interventionist approach, claiming that his gland grafting procedures—the hardest of hard regenerations—could overcome the chaotic decline of an organism even as complex as a human being.

Following the popularity of nerve tonics in the nineteenth century, the early twentieth century—most especially the period following WWI—saw the regeneration of nerves and the nervous system as a major source of attention, both biologically and commercially. Although neurasthenia had been recognised as a condition since the latter stages of the nineteenth century, the emergence of shell shock amongst those serving in the military gave new imperatives to consider how to restore faculties (Loughran, 2013; Loughran, 2012; Meyer, 2009; Micale, 2008). As well as a focus on individual bodies, improvement of society was a related goal of would-be regenerators in the early twentieth century. Eugenic ideals of a stronger race were rooted in the betterment of the individual, yet they also spoke to wider concerns about overpopulation as well as the onset of decrepitude in the aged.^3^

Regenerative concepts therefore span everything from the animal models which underpinned modern developmental physiology to the revivification of economies, urban spaces, the environment and fictional characters. The linguistic continuity across these domains serves to highlight the extent to which biological and social understandings of regeneration acted reciprocally. Implicit in regeneration discourse is the language of normalcy. To regenerate something implies that there is a defective, corruption or lessening to be corrected; it is the exact counterpoint to degeneration. Although regeneration as a biological process is hardwired into a range of creatures, from hydras to salamanders, in humans the concept has been extended to cover interventions in the form of biosynthetic scaffolds, prostheses, chemical stimulants, and other tools and techniques for rebuilding and restoring the body.

In contemporary biomedical science, however, 'regenerative medicine' is closely associated with bioengineering and molecular biology; it is linked to regenerating human cells, tissues or organs. Regenerative medicine encompasses a broad range of biomedical research and its clinical application on different scales, from stem cell manipulation to the transplantation of organs grown in vitro. However, regeneration in humans has been associated historically with numerous diverse therapies, from sleep and convalescence to electrotherapy and exercise. Regeneration itself is an ambiguous and wide-ranging term, with multiple context-dependent meanings. In the case of human beings it has come to refer to the restoration of bodily structure and function, implying a response to pathology or a loss of physiological function. Yet to adhere to such a perspective is to ignore the restorative, reparatory, and therapeutic dimensions of regeneration, all of which are active at an individual level.

### Towards new perspectives on human regeneration

The collection of articles^4^ accompanying this paper represents the first attempt to set out, interrogate and explain the diverse landscape of human regeneration, from everyday practices in skincare to representations of regeneration in recent film. By positioning attempts to regenerate the bodies and minds of humans within their wider social and cultural contexts, we learn more about the motivations driving the desire for regeneration, the role of commercial forces in monetising this yearning, the relationship between theories and practices of regeneration, and the historical and contemporary controversies surrounding its different forms. What does it mean to regenerate? How has biomedical knowledge shaped cultural representations of regeneration and degeneration? How have ‘natural’ means of regeneration—diet, sleep, exercise—functioned alongside medico-technical intervention? How has regenerative medicine changed our relationship with our bodies, beauty and ageing? In what contexts have different definitions of ‘regeneration’ been constructed? How do historical narratives of regeneration/degeneration interact with ideas about the hetero-normative body?

The papers explore these questions by interrogating some of the many meanings and practices of regeneration in a wide range of contexts, from clinical practice to popular culture and from Aristotelean perspectives on generation and growth of the human body to imagined futures of regenerative medicine. In the process, the papers both individually and collectively break important new ground by tracing how and why specific forms of regeneration become part of the public and professional medical landscape. Drawing on both historical and literary perspectives this exploration of regeneration advances our understanding of the human body and its associated medical practices by enhancing three major areas of historical scholarship.

First, the history of ageing and the body, exemplified in works by Hyung Wook Park, Ina Zweiniger-Bargielowska, Pat Thane, Kay Heath and others (Park, 2016; Zweiniger-Bargielowska, 2012; Heath, 2009; Thane, 2005). We know that ageing—increasingly conceptualised in Western society as a process of decline from the late nineteenth century—was a source of social anxiety and an inspiration for many attempts at human regeneration. This collection shows how the natural process of ageing—whether physiological or aesthetic—intersected with practices and theories of regeneration. Chris Gilleard shows how the concept of regeneration as understood in antiquity was incorporated into key medieval texts, which themselves show remarkable congruency with more recent manifestations of research into regeneration. In other cases, such as those instances from the late nineteenth century explored by Jessica P. Clark, new constituencies of would-be authority figures attempted to claim expertise and ownership over regenerative procedures. Elsewhere, as noted in the contributions of Catherine Oakley and Lucy Burke, narratives of regeneration played an important role in fictional reimaginings of our relationship with ageing, decline and normativity.

Second, the article collection draws on the historical relationship between medicine and commerce, such as the work of Takahiro Ueyama and Claire L. Jones, showing how medical practitioners, entrepreneurs and corporate interests attempted to promote products which promised to regenerate body and mind (Jones, 2013; Ueyama, 2010). These included specific foodstuffs and diets, as well as supplements and medical devices. Lucy Burke explores how the regeneration of minds in recent speculative fictions can inform a critique of ageing as a process understood in market terms. For Clark, the process of commodification was a central factor in shaping public discourse concerning regeneration; periodicals and publication strategies designed to maximise commercial gain from regenerative methods, most clearly for women, presented a new challenge for a medical profession who were themselves ambiguously associated with commerce. The wider context of developments in regenerative medicine and the importance of the relationship between biomedical and medical practice and the medical humanities forms the basis for the paper by Jennifer Edwards, Richard Thomas, and Robert Guilliatt.

Third, we know through studies in the history of gender that male and female bodies were conceptualised in very different ways. Examples of this include George L. Mosse’s foundational work on masculinity and Brigette Soland’s study of female identity (Soland, 2000; Mosse, 1996). The papers in the special issue provide a further layer to this research by showing how potentially universal (or at least universalisible) concepts of regeneration and regenerative capacity were applied to both bodies and minds of men and women. A particular instance of this forms the basis of Cheryl Logan’s article on the importance of endocrinology for a new form of plastic, holistic, neuropsychiatry which enjoyed a period of favour in early-twentieth-century Switzerland. The rejuvenation of men in the early twentieth century was seen to provide a particularly problematic moral case: was it socially acceptable or desirable to be enabling older men to return to the promiscuous habits of their youth?

Across its individual contributions, the collection encourages scholars in the medical humanities to explore the body as a medical, cultural and social construct and question its normativity. In the process the collection also highlights how perspectives from both literature and history can complement one another to illuminate in new ways the role of regeneration as both a medico-scientific and an imaginative vehicle for exploring fundamental aspects of what it means to be human.

Chris Gilleard explores the reciprocal interaction between theology, medicine and natural philosophy in the medieval world (Gilleard, 2017). Focusing closely on work of the thirteenth century physician and religious reformer Arnauld de Villanova, Gilleard shows the continuity and significance of Aristotelean theories of growth, decay and regeneration for de Villanova’s *Tractatus de Humido Radicali* (treatise on radical moisture). Villanova concluded that natural limits of longevity determined the extent to which organisms might be regenerated or restored. The propensity for organisms to regenerate depended on radical moisture which, over time, hardened and could not simply be replaced by the ingestion of food to promote growth. Radical moisture was an essential developmental component, deriving from conception, which was finite and provided a justification for the limitation of human regeneration.

Villanova and a number of his contemporaries, including his Montpellier colleague Bernard di Gordan, also provided readers with details of specific preparations calculated to maximise health which drew heavily on the non-naturals. Intriguingly, Gilleard argues that the confluence of ancient and medieval perspectives provided a critical basis for subsequent investigations into bodily regeneration and decay until the onset of the scientific revolution. However, even in an age of scientific medicine from the nineteenth century onwards, the majority of possibilities of rejuvenation blend hyperbole with aspects of organised investigation in a direct parallel of what Gilleard terms the ‘pre-modern precursors’ to the science of regeneration.

In the intervening centuries the search for youth came to encompass a still wider range of activities beyond simply restoring an earlier physiological state. Chief amongst these was the application of scientific ideas to promote an improved aesthetic. In her article ‘“Clever Ministrations”: Regenerative Beauty at the *Fin de Siècle*’, Jessica P. Clark provides a window into a hitherto neglected episode of regenerative beauty practices and products (Clark, 2017). Focusing primarily on the British case—at once distinctive but with clear resonance for other localities—Clark uses the figure of the 'beauty culturist' to explore how everyday regenerative practices, including the use of products decried in the medical press as ‘quack concoctions’, became a necessity rather than a curiosity. Driven by commercial imperatives, beauty culturists searched for legitimacy, a process followed eagerly through the periodical press where breakthroughs and (sometimes shocking) failures were reported extensively to public audiences.

For Clark, regenerative possibilities of the aesthetic variety created something of a sensation as previously hidden, domestic activities designed to promote youth—practised privately—became legitimate topics of public debate. The critical confluence of new technologies and mobilisation of print cultures, including strategic advertising and self-promoting quasi-textbooks, created new visibility for these highly-gendered everyday regenerative methods, which occupied a curious space between the medical and cosmetic.

Away from the domestic sphere, Cheryl Logan investigates the singular relationship between the emerging sciences of neurophysiology and endocrinology (Logan, 2017). In early-twentieth-century Switzerland, Logan shows, the use of techniques developed in the search to understand hormones and the so-called 'ductless' glands of secretion underpinned a neuroplastic understanding of diseases of the brain; neuropathological processes could be reversed and the mind restored. The chief object of Logan’s investigation—the Swiss physician Louis Rudolf (Rodolphe) Brun—operated at the intersection of neurology and endocrinology and also drew, surprisingly, on the psychoanalytic thinking of Sigmund Freud. Brun’s approach was deeply rooted in the disciplinary anxieties of the time and offered psychiatry the opportunity to escape from what both Logan and Makari term the ‘nihilism and degenerationism that had plagued’ the discipline’s infancy. Ultimately, Brun’s ‘Freudian neuroendocrine holism of the energetics of plastic engram networks’ was rendered obsolete by the emerging dominance of American-inspired reductionism in the immediate aftermath of World War II. Logan suggests a more pervasive anxiety surrounding degenerative approaches common to both scientific and social thought in the late nineteenth century. The same ideas in endocrinology which fanned a public fascination with rejuvenation in the interwar period were at the same time a critical factor in Brun’s attempt to establish a new approach to psychiatry.

Scientific discourse and public debate around the possibility of human regeneration were highly visible in the first half of the twentieth century. Especially in the early- to mid-1920s, this medico-social phenomenon inspired a particular sub-genre of fiction—the rejuvenation novel—which flourished, particularly in Britain and the United States. Novels such as Gertrude Atherton’s *Black Oxen* (1923) and Bertram Gayton’s *The Gland Stealers* (1922) drew on social apprehension by characterising rejuvenation as an ultimately doomed enterprise.^5^ Catherine Oakley shows how, even though the scientific ideas which underpinned these narratives had already fallen steeply out of fashion, the trope persisted beyond the short-lived high-water mark of rejuvenation fiction in the 1920s (Oakley, 2018). Her examination of the content and context of C. P. Snow’s anonymously published and commercially unsuccessful second novel, *New Lives for Old* (1933), reveals how rejuvenation was not only socially but also politically divisive. As Oakley highlights, Snow drew on his own experiences as a doctoral student to reconstruct the impact of the discovery a rejuvenation elixir at the level of both individual and nation. Drawing on previously-unknown archival material surrounding the book’s production, Oakley’s analysis provides further confirmatory evidence of the far-reaching impact of rejuvenation research on socio-cultural perspectives of ageing and youthfulness, as well as shedding new light on how rejuvenation was radically and damagingly gendered in accounts such as Snow’s.

Whilst the decades around 1900 arguably saw the emergence of regeneration as a significant object of study for the biological and biomedical sciences, its current manifestation—in the form of regenerative medicine—has become increasingly concretised and organised. Considering the current state of regenerative medicine and its origins in a range of disciplines across the sciences, Jennifer Edwards, Richard Thomas and Robert Guilliatt highlight the importance of reciprocal dialogue between the medical humanities and regenerative medicine (Edwards, Thomas and Guilliatt, 2017). Given the potential for hyperbolic acclamation of regenerative medicine and its potential impact on human medicine and health, they argue that a deeper understanding of ‘the historical context, ethical implications and public perception’ of the field is currently absent and has the potential to better situate laboratory innovations within both clinical practice and wider society. Indeed, as Edwards, Thomas and Guilliatt show, the hybrid nature of the field of regenerative medicine, with roots in cell biology, bioengineering and genetics amongst other disciplines, necessitates a fresh perspective on the potential socio-cultural impacts of a range of regenerative treatments being fully realised.

On the one hand, aspects of regenerative medicine might be integrated seamlessly into existing forms of clinical practice and specialism. On the other there might be inherent value in introducing regenerative medicine as an area of clinical practice as well as a biomechanical research field.^6^ Given the proliferation of different products and procedures which might eventually make their way into standard clinical encounters with patients it is prudent to approach this question from a historical perspective. For example, the fascination with strategies which promised life extension, improved health and even the prolongation of youth gives us reason to believe that any treatments labelled as ‘regenerative’ are likely to arouse increased optimism and interest on the part of patients. Managing the expectations around the field of regenerative medicine (and, indeed, in medical innovations more generally) is critical as developments move from the laboratory through clinical trials to the medical market, and finally to patients.

As well as underpinning new approaches to biomedical restoration of human function, conceptual applications of regeneration also yield some of the most potent vehicles for constructing imagined futures. By considering three examples from popular culture, predicated on the possibility of ‘neural regeneration’, Lucy Burke uncovers some of the fundamental instabilities associated with our perception of dementia—and ageing more broadly—as a problem which is conceived in market terms at the level of the individual (Burke, 2017). Burke’s use of science and speculative fiction demonstrates not only how considering imagined forms of regeneration can show the inadequacy of our current attitudes towards states associated with ageing, but also the significance of these cultural materials for challenging dominant views of the hetero-normative body. Indeed, it is a key assumption of regenerative narratives that there is a state—typified by 'full functionality'—towards which one is regenerated. Such representations serve to reinforce norms in ways which intersect with trends in disability studies traceable to the foundational work of Lennard J. Davis and others on normalcy (Davis, 2014; Davis, 1995). Ultimately, Burke concludes, fully-realised regenerative outcomes in fiction enable us to better see how we might, in reality, come to terms with the necessarily incomplete consequences of regenerative therapies for conditions which are themselves progressively degenerative in nature.

### Presents and futures of 'medical' regeneration

The field of regenerative medicine in the contemporary world might be a heterogeneous one as far as bio-clinical disciplines are concerned, yet our appreciation of its multifaceted roots remains largely incomplete. The essence of regenerative medicine lies in the replacement of defective parts of the human body with analogues, which might be composed of anything from tissues grown *in vitro* to scaffolds and mechanical systems. Whatever the nature of the intervention, it is clear that the ‘regenerative’ aspect of regenerative medicine applies to the organism as a whole rather than a specific system, organ or tissue, which is in fact replaced or significantly modified. Indeed, I would argue, the actual ‘regeneration’ taking place is one of function, not structure. In this sense, regenerative medicine is as much about restoration and repair as it is about actual regeneration, a term which is arguably laden with greater promise. As noted in a major report on regenerative medicine by the UK Government Select Committee on Science Technology, ‘[r]egenerative medicine provides a unique approach to treating diseases and disorders by providing the body itself with the means to repair, replace, restore and regenerate damaged or diseased cells, tissues and organs’ (Science and Technology Committee, 2017).

Goethe’s vision of regeneration, articulated in the late eighteenth century, drew on ancient biological understandings of renewal and were refracted through contemporary practices of natural philosophy. The questions which it raised are still being continually reshaped by modern biomedicine, yet at their heart they remain fundamentally unchanged and just as significant. Knowing more about the use of regenerative ideas in science, society and culture provides an opportunity to interrogate more deeply the human desire for restoration. The papers presented here illuminate selected examples of regenerative thought, concentrating on the transformative period around 1900, but much more remains to be done. How, for example, were ideas and narratives of regeneration applied beyond the biological realm? In what ways has resistance to regeneration been manifested? To what extent does the term ‘regenerative’ transform patient and public expectations of medical science and clinical practice? What aspects of 'hard' and 'soft' regeneration are identifiable across the range of practices, from the laboratory and clinic to the home?

These questions and others provide motivation for these papers and for further inquiry into the nature of regenerative processes and their relationship with humans. Indeed, as we are surrounded by positive narratives about the benefits and desirability of regeneration—of our bodies, lives and our urban and social spaces—this might be an opportune moment to reflect on exactly why it is that 'regeneration' is held up in such terms. An interdisciplinary approach to this topic—which mirrors the large body of extant scholarship on degeneration—might help us to better understand its attractions, problems and controversies.

## Notes

1 A limbeck, otherwise known as an alembic, was an alchemical vessel used for distilling liquids.

2 Fink describes Goethe’s science as ‘a narration of the acts of nature as he found them in the transformation of rocks, in the linkage of bones, in the transitions of the leaf, and in the generation of colours’ (Fink, 1991, p 50). Much scholarship in historical and literary studies has focused on *Faust*, although historians of science and medicine have not engaged wholesale with this work. In the field of literature and science, the classic work of Roslynn D. Haynes has illuminated the significance of ‘the scientist’ in this and other works of fiction. See: Haynes (1994).

3 Numerous pamphlets and other publications dedicated to eugenic forms of regeneration appeared in the aftermath of the First World War when national fitness and economic security became pressing matters in the face of the potential for global conflict.

4 Human Regeneration collection (2018), *Palgrave Communications*: https://www.nature.com/collections/yxtkjcdxbr

5 Later examples include M. E. Mitchell’s *Yet in my Flesh* (1933), John Gloag’s *Winter*’*s Youth* (1934), Thorne Smith’s *The Glorious Pool* (1934), and Aldous Huxley’s *After Many a Summer* (1939).

6 For more on the discussion of translating regenerative medicine into a clinical context, see in particular outputs associated with the REGenableMED project, such as: Gardner and Webster (2016); Faulkner (2016); Gardner and Webster (2015).
